# The Evolving Treatment of Peripheral Arterial Disease through Guideline-Directed Recommendations

**DOI:** 10.3390/jcm7010009

**Published:** 2018-01-09

**Authors:** Ramez Morcos, Boshra Louka, Andrew Tseng, Sanjay Misra, Robert McBane, Heidi Esser, Fadi Shamoun

**Affiliations:** 1 Department of Internal Medicine, Florida Atlantic University, Boca Raton, FL 33431, USA; ramezmorkous@gmail.com; 2 Department of Cardiovascular Diseases, Mayo Clinic, Scottsdale, AZ 85259, USA; louka.boshra@mayo.edu (B.L.), tseng.andrew@mayo.edu (A.T.); 3 Department of Cardiovascular Diseases, Mayo Clinic, Rochester, MN 55905, USA; misra.sanjay@mayo.edu; 4 Department of Radiology, Mayo Clinic, Rochester, MN 55905, USA; mcbane.robert@mayo.edu; 5 Arizona Center for Urology, Phoenix, AZ 85027, USA; heidi.esser.85@gmail.com

**Keywords:** peripheral arterial disease, guidelines, claudication, ankle brachial index

## Abstract

Peripheral arterial disease (PAD) refers to partial or complete occlusion of one or more non-coronary arteries that leads to compromised blood flow and ischemia. Numerous processes are involved in arterial stenosis, however, atherosclerosis remains the most common etiology. PAD constitutes a major health economic problem, and it is estimated that over 200 million people around the world suffer from PAD, with at least 20% having some degree of claudication. The purpose of this review is to compare and contrast the guidelines on PAD published in 2005, 2011 and 2016 in terms of new recommendations and level of evidence for practicing clinicians.

## 1. Introduction

Peripheral arterial disease (PAD) refers to partial or complete occlusion of one or more non-coronary arteries that leads to compromised blood flow and ischemia [1]. Numerous diseases may impair blood flow to lower extremity arteries, however, atherosclerosis remains the most common process of the disease. Risk factors for PAD include age, diabetes mellitus, smoking, renal disease, hyperlipidemia, hypertension and other non-traditional risk factors, i.e., ethnicity, metabolic disorders, and C-reactive protein (CRP) [2]. According to one estimate, PAD affects more than 200 million individuals worldwide [3,4]. In another estimate, 14% of the population over the age of 70 suffers from PAD, with the disease affecting men and women equally [5]. The Systolic Hypertension in the Elderly Program has reported a higher incidence of PAD in African American males than in females [6]. The most common symptom of PAD is intermittent claudication: leg pain on walking that improves with short rest [2,3]. Although the typical symptom of PAD is classic claudication, this symptom may present in only 11% of cases [7]. PAD poses a significant economic burden to many countries. For example, in United States, PAD increased the number of hospital admissions, costing $6000 per patient per year [8].

The American College of Cardiology (ACC) and American Heart Association (AHA) Task Force on Practice Guidelines develops and revises practice guidelines, and writing committees comprising independent authors representing both the ACC and AHA develop written manuscripts for practice guidelines [7].

**Figure 1:**
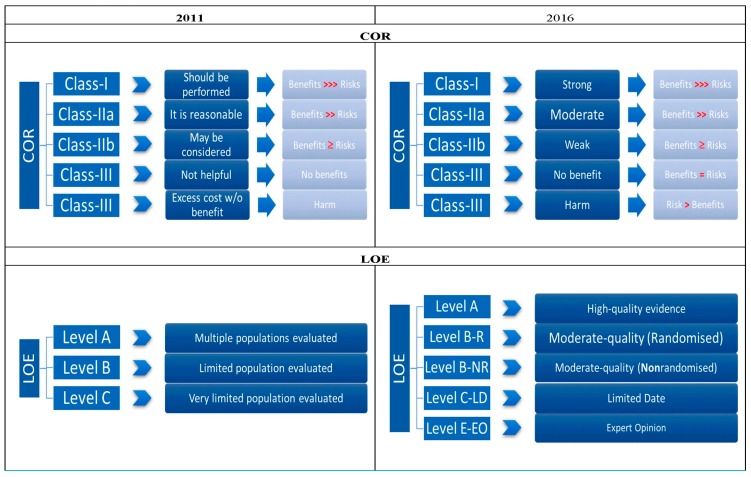
Comparison between guidelines 2011 and 2016 in terms of class of recommendations (COR) and level of evidence (LOE).

Medical science is evolving, similarly, the management of PAD continues to change on the basis of current trials and advanced diagnostic and treatment strategies. The purpose of this review is to compare and contrast the ACC/AHA PAD guidelines published in 2005, 2011 and 2016 in terms of evolving recommendations and levels of evidence (Figure 1).

## 2. Guidelines

### 2.1. History and Physical Exam

The 2005 guidelines recommended obtaining a history of falls, leg weakness, walking impairment, claudication, ischemic rest pain, and non-healing wounds of patients greater than 50 years old with risk factors for atherosclerosis or of all patients greater than 70 years old. It was suggested that the ankle-brachial index (ABI) and toe-brachial index might be helpful for the diagnosis of PAD in asymptomatic patients at risk. The 2011 guidelines, in terms of history and physical examination, were the same except the emphasis on performing ABI to establish the diagnosis of lower extremity PAD in those with exertional leg symptoms, non-healing ulcers, age greater than 65 years, or age greater than 50 years with a history of smoking or diabetes. The level of evidence increased from (C) to (B) [9]. The 2016 guidelines further stressed the value of a complete vascular examination (inspection, palpation and auscultation) and the measurement of non-invasive blood pressure in both arms at least at an initial visit [10,11]. It is important to address all vascular risk factors such as atherosclerosis and hypertension because they significantly contribute to PAD development [12]. The level of evidence changed from (I C) in 2005 to (I B-NR) in 2016.

### 2.2. Patients at Risk

According to the 2005 guidelines, smoking, hypertension, hyperlipidemia, diabetes and metabolic syndrome were considered the major risk factors for PAD. Age was also seen as a risk factor and was categorized as less than 50 years, 50–69 years and greater than 75 years. The 2016 guidelines modified the age categories to less than 50 years, 50–64 years and greater than 65 years. They further stated that risk factors for PAD included age greater than 65 years or age 50–64 years plus one risk factor for atherosclerosis. The decrease in age (from greater than 75 years in 2005 to greater than 65 years in 2016) was made in an attempt to diagnose PAD earlier. In other words, care providers should consider looking for PAD in individuals at age 65 or older as part of a routine vascular examination (Table 1).

### 2.3. Diagnostic Testing

The ankle brachial index (ABI) is reported to be a >90% sensitive and specific non-invasive tool compared to Angiography. It is a cost-effective, diagnostic as well as prognostic tool for PAD [13]. In 2005, ABI was recommended for asymptomatic lower extremity PAD, claudication and pseudo-claudication. The 2011 guidelines recommended that ABI should be used to establish PAD in individuals with symptoms suggestive of lower extremity PAD such as: exertional leg symptoms, non-healing wounds, age 65 years and older, or age 50 years and older with a history of smoking or diabetes. These recommendations were made at level of evidence (IB) and also added a borderline category to the levels of ABI abnormality. In 2016, the guidelines recommended that ABI, with or without segmental pressures and waveforms, should be used to establish the diagnosis in patients with clinical signs or symptoms suggestive of PAD, with a level of evidence of (IB-NR). However, in patients without clinical clues suggestive of PAD, the ABI is not recommended (II B-NR). Performing an ABI and leg segmental pressure measurements with an existing diagnosis of PAD remained at the same level of evidence from 2011 to 2016 (IIa). Guidelines regarding performing pulse volume recordings, leg segmental pressure, and continuous-wave Doppler ultrasound, remained unchanged in 2016, however, toe-brachial index was recommended to diagnose patients with suspected PAD when the ABI is >1.40. The level of evidence was changed from (IB) in both 2005 and 2011 to (IB-NR) in 2016.

Additionally, in the 2016 guidelines there were four more changes regarding the utility of physiologic testing with level of evidence (IIa B-NR): (1) in patients with normal (1.1–1.39) or borderline (0.91–1.09) ABI with tissue loss or gangrene, it is reasonable to diagnose critical limb ischemia (CLI) by using tissue oxygen pressure and tissue perfusion TcPO_2_, or toe brachial index (TBI) with waveforms; (2) in patients with PAD, i.e., an abnormal ABI (≤0.90), or with non-compressible arteries (ABI > 1.40 and TBI ≤ 0.70) with non-healing wounds or gangrene, TBI with waveforms, TcPO_2_ can be useful to evaluate local dermal perfusion (3) patients with established PAD should be considered for exercise ABI testing to assess functional status accurately [14]; and (4) patients with exertional leg symptoms (not related to arthritis or neurogenic claudication) and normal or borderline resting ABI (0.9–1.1) should undergo exercise ABI testing to assess for the presence of significant PAD.

Duplex ultrasound (DUS), computed tomography angiography (CTA), or magnetic resonance angiography (MRA) with or without gadolinium of the lower extremities is useful to locate the area and severity of arterial stenosis in symptomatic patients who might be considered for revascularization [15]. In the 2005 guidelines, DUS was recommended for routine surveillance after surgical revascularization with either femoral-popliteal or femoral-tibial bypass with a venous conduit, and it was suggested as a useful tool for the selection of candidates for endovascular intervention. Similarly, CTA and MRA were recommended for use in locating the area of stenosis in patients with PAD. No modifications were made in this area in 2011. However, in the 2016 guidelines, DUS, CTA and MRA of the lower extremities were all considered useful in addressing the anatomy and degree of stenosis in symptomatic patients prior to revascularization (I B-NR).

Regarding invasive imaging in the 2005 and 2011 guidelines, digital subtraction angiography was recommended for contrast angiographic studies to better visualize the arterial flow, and was suggested for imaging of the iliac, common femoral, superficial femoral and tibial arteries without vessel overlap. In 2016, invasive angiography was recommended for patients with CLI in whom revascularization is being considered (I C-EO). Invasive angiography and revascularization was reasonable for patients with lifestyle-limiting claudication with an inadequate response to medical therapy (IIa C-EO) [16] (Table 2).

### 2.4. Screening

In the 2005 and 2011 guidelines, no screening for other vascular diseases was recommended for PAD patients, however, in the 2016 guidelines screening ultrasound for abdominal aortic aneurysm (AAA) was recommended in individuals with symptomatic PAD (IIa B-NR) [17,18]. DUS is an inexpensive, accessible, and accurate screening tool for undiagnosed AAA [19]. Currently, there is no evidence to suggest that screening for PAD in asymptomatic patients with atherosclerosis in other arterial beds impacts clinical outcomes. In spite of this, the 2016 guidelines recommend evaluating the patient at elevated risk for atherosclerosis under these conditions: (a) age 65 years or older, (b) age 50–64 years with diabetes or history of smoking or hyperlipidemia or hypertension or family history of PAD, (c) age 50 years, or older, with diabetes and other risk factors for developing atherosclerosis, and (d) patients with evidence of atherosclerotic disease in other vascular beds.

### 2.5. Medical Management

Medical therapy for PAD includes polypill drug such as antiplatelet, antihypertensive, statins, angiotensin converting enzyme inhibitors, angiotensin receptor blockers, diabetic medication, and Cilostazol. The AHA/ACC 2005 guidelines recommended Aspirin 75 to 325 mg per day as a safe and effective antiplatelet agent (IA) with Clopidogrel (75 mg per day) as an alternative antiplatelet therapy to aspirin (IB). These recommendations were revised in 2011 with two new recommendations and further clarification on symptomatic PAD. The 2011 guidelines also changed the level of evidence for aspirin dosing from (IA) to (IB). It is recommended that antiplatelet therapy be used to reduce the risk of MI, stroke, or vascular death in asymptomatic individuals with an ABI less than 0.90 (IIa C) while the efficacy for aspirin therapy to prevent limb ischemia in asymptomatic individuals with borderline abnormal ABI, defined as 0.91 to 1.09, is not well established (IIb A). Similarly, the guidelines recommended that the combination of aspirin and clopidogrel could be considered to reduce the risk of cardiovascular events in patients with PAD whose bleeding risk is low and who are at high cardiovascular risk, including those with prior lower extremity revascularization (endovascular or surgical), intermittent claudication or critical limb ischemia, or prior amputation for limb ischemia (IIb B). In the 2016 guidelines, in asymptomatic patients with PAD (ABI ≤ 0.90), antiplatelet therapy was an acceptable choice to lower the risk of myocardial infarction and stroke (IIa C-EO). Similarly, in asymptomatic patients with borderline ABI, i.e., 0.90–0.99, the efficacy of antiplatelet therapy to reduce the risk of cardiovascular death is uncertain and it may increase the risk of bleeding (IIb B-R). Moreover, the efficacy of dual antiplatelet therapy (DAPT) to lower cardiovascular events in patients with symptomatic PAD has been questioned in the presence of elevated bleeding risk, as it is not well established (IIb B-R). In this regard, two new recommendations were made in 2016: (1) DAPT may be reasonable to reduce the risk of limb ischemia or gangrene in high risk patients with symptomatic PAD who underwent a lower extremity revascularization procedure (IIb C-LD) [20]; and (2) in the TRACER trial Vorapaxar showed modest decrease in mortality when combined with aspirin in patients with symptomatic PAD, however, that was offset by a moderate to severe increased risk of bleeding in patients with PAD (hazard ratio 1.47, 95% CI 0.89–2.45) and patients without PAD (hazard ratio 1.48, 95% CI 1.22–1.79; *p* interaction = 0.921) (IIb B-R). Stain therapy is indicated in all three set of guidelines. However, the level of evidence changed from I B to I A in 2016. Additionally, contrary to the 2005 guidelines, fibrate derivatives are not recommended in the 2016 guidelines.

Antihypertensive therapy is recommended in all three guidelines to patients with hypertension and PAD to reduce the risk of cardiovascular events and heart failure, with an unchanged level of evidence (IA). In 2016, the use of angiotensin-converting enzyme inhibitors (ACEIs) or angiotensin-receptor blockers (ARBs) is recommended to reduce the risk of cardiovascular events in patients with established PAD, with the level of evidence changed from (IIa B) to (IIa A) in 2016. The treatment with ACEIs for asymptomatic PAD patients was recommended with evidence (IIb C) in 2011, however, they were not recommended for the same in 2016.

Smoking cessation is recommended in all three guidelines with different level of evidence: (I B) in 2005, (I C) in 2011 and (I A) in 2016. The 2011 guidelines recommended counseling for tobacco cessation (I A) and anti-smoking agents at every visit (I A). The 2016 guidelines recommended that patients with PAD avoid exposure to second hand smoking at home and in public places (IB-NR) [21].

In regards to glycemic control, the 2005 and 2011 guidelines recommended a target HbA1c <7%, however, the 2016 guidelines did not have an A1c target (IIa C) and furthermore recommended that the management be determined by members of the healthcare team (I C-EO). Moreover, they added that good glycemic control can be beneficial for patients with CLI to reduce tissue loss and amputation (IIa B-NR).

Anticoagulation has been discouraged in all three guidelines with different levels of evidence, i.e., (III C) in 2005, (III B) in 2011 and (III A) in 2016. In the 2016 guidelines the efficacy of anticoagulation to improve autogenous vein or prosthetic graft patency after lower extremity bypass was uncertain (IIb B-R). The results of the COMPASS trial are not published at the time this manuscript is being written.

Cilostazole is recommended in the 2016 guidelines as was also recommended in those in 2005 and 2011, however, the use of pentoxifylline was discouraged in the 2016 guidelines. Another new recommendation in the 2016 guidelines for patients with PAD was an annual influenza vaccination (I C-EO) [22].

### 2.6. Supervised Exercise

A supervised exercise program is considered first-line therapy for claudication. Evidence shows that exercise training not only benefits those with PAD and claudication but also helps those without claudication. Supervised exercise training (walk-program) was recommended as an initial treatment modality for patients with intermittent claudication (IA) in the 2005 guidelines. The CLEVER study showed the change in peak walking time was greatest with supervised exercise, intermediate with stenting and least with optimum medical care (OMC) (mean change versus baseline, 5.8 ± 4.6, 3.7 ± 4.9, and 1.2 ± 2.6 min, respectively; *p* < 0.001) [23,24].

According to the 2016 guidelines, in patients with walking impairment, a supervised exercise program should be recommended to improve quality of life (QoL), increase functional status and reduce claudication (IA). Three new recommendations have been added regarding supervised exercise: (1) a supervised exercise therapy (SET) should be considered as a treatment option for claudication before surgical or endovascular revascularization (I B-R); (2) a home or community based exercise program with life-style modification can improve walking ability and functional status (IIa A); and (3) in symptomatic patients who cannot enroll in SET, alternative forms of exercise should be considered including cycling, upper-body ergometry, and low-intensity walking, which strategies can be beneficial in improving functional status and walking ability (IIa A).

### 2.7. Foot Care and Minimizing Tissue Loss

In the 2016 guidelines patients with PAD and diabetes should be counseled by the health care team about healthy foot care and self–foot examination. Prompt diagnosis and treatment of foot wounds are recommended to avoid limb gangrene. In patients with PAD and foot ulcerations, prompt referral to a multi-specialty wound-care team should be considered. It is also reasonable to counsel non-diabetic patients with PAD about self–foot care. Biannual foot examinations by a health care provider is reasonable for patients with PAD [25].

### 2.8. Revascularization for Claudication

Endovascular techniques include revascularization with angioplasty, stents, and atherectomy. The revascularization techniques and atherectomy devices continue to involve, Criqui and Aboyans [1] in his EXCITE ISR prospective randomized trial demonstrated superiority of excimer laser atherectomy plus percutaneous transluminal angioplasty (PTA) versus PTA alone for treating in-stent restenosis of the femoropopliteal artery. The three sets of guidelines recommended revascularization for individuals with a vocational or lifestyle-limiting disability due to lower extremity claudication who had an inadequate response to exercise and pharmacological therapy (IIa A). Endovascular revascularization was recommended as an effective option for patients with symptomatic claudication and significant aortoiliac (inflow) occlusive disease (IA). Endovascular procedures are an effective option for patients with symptomatic lower extremity claudication and significant femoral or popliteal disease (IIa B-R). The efficacy of endovascular procedures for patients with claudication due to isolated infra-popliteal artery disease is unknown (IIB C-LD). Endovascular procedures should not be performed in asymptomatic patients to prevent progression disease progression to CLI (III-B-NR).

### 2.9. Critical Limb Ischemia

Critical limb ischemia (CLI) refers to the condition where ischemic rest pain, non-healing ulcers or tissue loss in one or both legs remains for two or more weeks, and the condition is objectively attributable to proven arterial occlusive disease. The 2005 guidelines, recommended that invasive therapeutic interventions be considered after complete anatomic assessment of the affected arterial territory, including imaging of the occluding lesion along with the assessment of arterial inflow and outflow with angiography. In the 2011 guidelines, two additional recommendations were added: (1) for patients with critical limb ischemia and an elevated operative risk and estimated life expectancy of two years or less, revascularization with balloon angioplasty is reasonable to perform when possible as the initial procedure to improve lower extremity blood flow (IIa B), and (2) for patients with limb-threatening ischemia and an estimated life expectancy of more than two years, surgical revascularization with an a vein conduit is acceptable to perform as the initial treatment to improve lower extremity blood flow (IIa B). In the 2016 guidelines, endovascular procedures for in-line blood flow (IB-R), use of a staged approach to endovascular procedures (IIa C-DL), evaluation of lesion characteristics (e.g., length, anatomic location and extent of the lesion) (IIa B-R), use the concept of angiosome-directed endovascular intervention for wound healing and limb salvage (IIb B-NR), bypass to the popliteal or infra-popliteal arteries with autogenous vein during surgery for CLI as compared to prosthetic graft material (IA), surgical procedures for in-line blood flow (IC-LD), prosthetic material for failed endovascular revascularization (IIa B-NR), and use of a staged approach for surgical procedures (IIa C-LD) have all been encouraged and recommended.

The 2016 guidelines recommended a comprehensive plan when treating CLI with revascularization and wound healing which will require interdisciplinary care team. The care team should achieve complete wound healing and a functional foot (I B-NR), the goal of wound care after revascularization is complete wound healing (I C-LD), intermittent pneumatic compression could be used for augmenting wound healing and/or severe ischemic rest pain (IIb B-NR), the effectiveness is still unknown for the use of hyperbaric oxygen therapy for wound healing (IIb C-LD), and the use of Prostanoid is not indicated when treating patients with CLI and considered with no benefit.

### 2.10. Acute Limb Ischemia

This is also one of the new items in the AHA/ACC 2016 guidelines. Patients with acute limb ischemia (ALI) must be urgently examined by a vascular specialist to assess limb viability and initiate expedited treatment (I C-EO). In individuals with ALI, systemic anticoagulation with heparin must be started as soon as possible (I C-EO). Etiology and degree of ischemia should be used to determine the revascularization strategy (I C-LD). Catheter-based thrombolysis is recommended for individuals with ALI (I C-LD) [26]. Patients should be observed for possible perfusion ischemia and compartment syndrome after revascularization (I C-LD). Percutaneous thrombectomy can be added to thrombolysis for patients with ALI who have salvageable limb (IIa B-NR). In individuals with ALI secondary to an embolism, surgical thrombo-embolectomy can be useful (IIa C-LD). The use of ultrasound for catheter based thrombolysis still unknown when used for individuals with ALI with a salvageable limb (IIb C-LD). In addition, testing for a cardiovascular source is recommended (IIa C-EO) [26].

### 2.11. Longitudinal Follow-Up

This is a new recommendation in the 2016 guidelines to consider an annual follow up for PAD patients to address cardiovascular risk factors, limb symptoms and functional status (I C-EO). Duplex ultrasound can be used for follow up in patients with a history of infra-inguinal bypass grafts (IIa B-R) (Table 3).

## 3. Conclusions

This is a succinct comparative review of recent guidelines (AHA/ACC 2016) on the management of PAD and the previous sets of guidelines (AHA/ACC 2005 and 2011) [7,27], Other contributions have been made by the society of vascular surgery and the inter-society consensus for the management of PAD (TASC II document) [28]. The recent recommendations have emerged with different level of evidence in the areas of history and physical examination, diagnostic testing, and management. The writing committee affirmed the importance of ABI in diagnosing PAD. It also emphasized the role of a supervised exercise program in the management of symptomatic patients and called for incorporating patient centered outcomes in future studies designed to test new endovascular treatments. Additionally, the new guidelines have discouraged the use of pentoxifylline, chelation, and homocysteine. The 2016 guidelines uncovered several knowledge gaps and areas of research, such as the need for new claudication medications and the need to determine the effect of diet on the development and treatment of PAD.

## Figures and Tables

**Table 1:** Comparison between guidelines 2005, 2011 and 2016 in terms of history, examination and risk factors for PAD.

Disease Aspects	2005	2011	2016	Comments
History + Examination	*Required:* Walking impairment, ischemic rest pain, and non-healing wounds.	Focused updates remained same.	*Additions:* Vascular examination for the patients with increased risk of PAD. Non-invasive BP measurements in both arms of the patients with PAD.	LOE changed from (IC) in 2005 to (IB-NR) in 2016. Two new recommendations with LOE of (IB-NR) were added.
Risks	Age (<50, 50–69, >70 years), leg symptoms with exertion, abnormal lower extremity pulse exam & K/C of atherosclerosis.	Focused updates remained same.	*Modifications:* Age (<50, 50–64, ≥65 years), K/C of atherosclerosis in another vascular bed or AAA.	The age categories were modified in terms of risk for the patients.
Screening	ABI can be used for PAD screening.	Focused updates remained same.	Screening DUS for symptomatic AAA.	A new recommendation was added.

*Abbreviations:* PAD: peripheral arterial disease; BP: blood pressure; LOE: level of evidence; K/C: known case of; AAA: abdominal aortic aneurysm; DUS: duplex ultrasound.

**Table 2:** Comparison between guidelines 2005, 2011 and 2016 in terms of diagnostic tests.

Test	2005	2011	2016	Comments
ABI	ABI with segmental pressures for PAD in both legs, without categorization; LOE (C).	ABI for exertional leg symptoms, non-healing wounds and risk factors for atherosclerosis. ABI results categorized. Other focused updates remained same.	ABI & segmental leg pressure for patients with presentation suggestive of PAD. Resting ABI recommended for patients with increased risk of PAD.	LOE changed from (IB) for ABI and segmental leg pressure, and ABI categories in 2011 to (IB-NR) and (IC-LD), respectively, in 2016. A new recommendation was added with LOE (II 1B-NR).
Physiological testing	Pulse volume recordings (IIa B), leg segmental pressure (IB), continuous-wave DUS (IB) for location and severity, and TBI for patients in whom ABI is not reliable. Exercise treadmill test for claudication and response to therapy. Pre- and post-exercise ABI to differentiate between arterial & non-arterial claudication.	Focused updates remained same.	TBI for suspected PAD with ABI > 1.40. TBI with wave-form, TcPO_2_ for normal, borderline ABI with non-healing wounds or gangrene to diagnose CLI. TBI with wave-form, TcPO_2_ or SPP for abnormal ABI, ABI > 1.40 and TBI ≤ 0.70 with non-healing wounds or gangrene. Exercise treadmill test for patients with walking impairment and normal or abnormal ABI. ABI before and after treadmill test for patients with PAD and abnormal resting ABI.	LOE changed from (IB) in 2005 to (IB-NR) in 2016 for TBI. Two new recommendations for ABI with wave-form added (IIa B-NR). Old recommendation for exercise treadmill test modified in 2016. A new recommendation added for exercise treadmill test.
Imaging	*Non-Invasive:* DUS for extremities for location and degree of stenosis (IA), endovascular intervention (IIB), and surveillance of femoral-popliteal bypass. CTA for anatomical location and the degree of stenosis in lower extremities in PAD patients (IIb B) and as a substitute for MRA (IIb B). MRA for anatomical location and degree of stenosis in PAD patients (IA) with gadolinium enhancement (IB) as well as for the selection of endovascular intervention (IA) and post-vascularization surveillance (IIb B).	Focused updates remained the same	DUS, CTA, and MRA of lower extremities for location and severity of stenosis in symptomatic patients with PAD.	A new recommendation added for imaging (IB-NR).
	*Invasive:* DSA for contrast angiographic studies (IA), and decisions for invasive therapeutic interventions (IB). Hydration for patients undergoing contrast angiography (IB). Follow-up for contrast angiography within two weeks (IC).		Angiography for patients with CLI in whom revascularization is considered (I C-EO). Angiography for patients with lifestyle-limiting symptoms with sub-optimal response to medical therapy (IIa C-EO).	New guidelines added (IC-EO & IIa C-EO).

PAD: peripheral arterial disease; LOE: level of evidence; K/C: known case of; DUS: duplex ultrasound; ABI: ankle brachial index; TBI: toe-brachial index; CLI: critical limb ischemia; TcPO_2_: transcutaneous oxygen pressure; SSP: skin perfusion pressure; CTA: computed tomography angiography; MRA: magnetic resonance angiography; GDMT: guideline-directed management and therapy.

**Table 3:** New additions in 2016 guidelines for PAD.

Disease Aspects	New Additions
Structured exercise	Supervised exercise program for claudication before revascularization (IB-R) Non-supervised exercise program with behavioral change techniques to improve functional status and walking parameters (IIa A) Upper-body exercises, cycling, and low-intensity walking are reasonable alternatives for patients with impaired ability to walk (IIa A)
Minimizing tissue loss in patients with PAD	Foot examination and foot care for PAD patients with or without DM including prompt medical attention for signs or symptoms of infections to avoid amputation Multidisciplinary care team for the treatment of foot infection Biannual foot examination by a medical provider
Acute limb ischemia	Emergent evaluation by a vascular specialist to assess limb viability and apply appropriate treatment (I C-EO) Rapid evaluation to assess for limb salvage (I C-LD) Initiation of parenteral anticoagulation with unfractionated heparin (I C-EO) Consideration of catheter-based thrombolysis for patients with ALI, a salvageable limb, and low risk of bleeding (I C-LD) Monitoring of patients for possible revascularization ischemia and development of compartment syndrome (I C-LD) Consideration of percutaneous mechanical thrombectomy as an adjunctive therapy to thrombolysis for patients with ALI (I C-LD) Consideration of surgical thromboembolectomy for patients with ALI due to an embolism with a salvageable limb (IIa C-LD)
Longitudinal follow-up	Return visits for clinical examination, including assessment of cardiovascular risk factors, limb symptoms, and functional status (I C-EO) Duplex Ultrasound for routine surveillance after surgical bypass grafts (IIa B-R)
Influenza vaccine	Influenza vaccination should be considered for all patients with PAD (I C-EO)

PAD: peripheral arterial disease; DUS: duplex ultrasound; ALI: acute limb ischemia; DM: diabetes.
